# Fast Potentiometric Analysis of Lead in Aqueous Medium under Competitive Conditions Using an Acridono-Crown Ether Neutral Ionophore

**DOI:** 10.3390/s18051407

**Published:** 2018-05-03

**Authors:** Ádám Golcs, Viola Horváth, Péter Huszthy, Tünde Tóth

**Affiliations:** 1 Department of Organic Chemistry and Technology, Budapest University of Technology and Economics, P.O. Box 91, H-1521 Budapest, Hungary; agolcs@mail.bme.hu (Á.G.); huszthy@mail.bme.hu (P.H.); 2 MTA-BME Computation Driven Chemistry Research Group, P.O. Box 91, H-1521 Budapest, Hungary; vhorvath@mail.bme.hu; 3 Institute for Energy Security and Environmental Safety, Centre for Energy Research, Hungarian Academy of Sciences, P.O. Box 49, H-1525 Budapest, Hungary

**Keywords:** electrochemical sensor, lead detection, crown ether, molecular recognition, ion-selective membrane

## Abstract

Lead is a particularly toxic heavy metal that is present above acceptable levels in the water of many countries. This article describes a quick detection method of lead(II) ions using a polyvinyl chloride (PVC)-based ion-selective membrane electrode containing an acridono-crown ether ionophore by potentiometry. The electrochemical cell exhibits a Nernstian response for lead(II) ions between the concentration range of 10^−4^ to 10^−2^ M, and can be used in the pH range of 4–7. The applicability of this sensor was verified by measuring a multicomponent aqueous sample. Under the given conditions, this electrode is suitable for the selective quantitative analysis of lead(II) ions in the presence of many additional metal ions.

## 1. Introduction

Lead is a widely used heavy metal. Its use extends to acid batteries, solder, alloys, cable sheathing, pigments, rust inhibitors, ammunition, glazes, and plastic stabilizers [1], so we must pay close attention to its effects on the environment and living organisms. In terms of drinking water, the pipes of plumbing systems contribute to a great degree of contamination [2,3].

Although the neurotoxicity of lead and its adverse effects on almost every organ in the body have long been known [4,5], many people in developing countries are exposed daily to poisoning due to water contamination. In untreated wastewater, the lead(II) ion could be present in dangerously high concentrations over 100 ppm [6,7].

Many spectroscopic methods have been developed so far for heavy metal detection [8,9,10,11,12,13]. These days, inductively coupled plasma-based optical and mass spectroscopic techniques are the most commonly used methods for simultaneous multi-element analysis. Although these methods provide unparalleled sensitivity and low detection limit under laboratory conditions, they require complicated instrumentation and specially trained personnel. People in developing countries are forced to monitor the quality of water regularly; therefore, small, portable, user-friendly equipment is needed.

**Figure 1:**
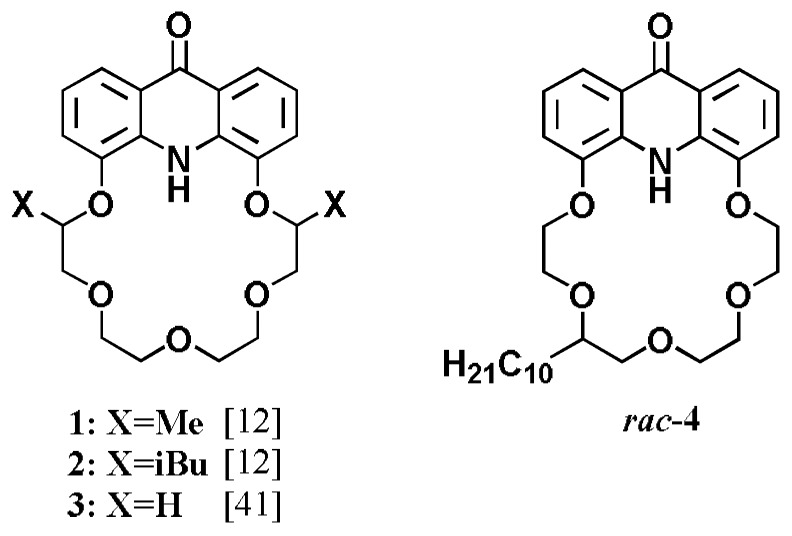
Macrocycles (**1**–**3**) possessing lead(II) ion selectivity and the newly synthesized ionophore *rac*-**4**.

Chemical sensors offer the opportunity for an easy, fast, and selective analysis of a single chemical species. In the perspective of the above applications, the development of new chemical sensors is still relevant today. Piroxicam, pyrophosphate, amide, carboxamide, benzoic acid, sulfide derivatives [14,15,16,17,18,19,20,21,22,23,24] and different types of supramolecular recognition agents (porphyrins, calixarenes, etc.) [25,26,27,28,29] have been efficiently used in several cases as electroactive sensing materials in lead detection. The largest number of lead-selective ionophores found in the literature belongs to the family of crown ethers [30,31,32,33,34,35,36,37,38,39,40]. In previous studies, acridono-crown ether derivatives (**1**, **2** and **3**, see Figure 1) were found to be highly selective for lead(II) ions due to the presence of a rigid, heteroaromatic tricyclic ring [12,41]. We were motivated to synthesize a new, lead-selective acridono-crown ether-type neutral ionophore, which could be incorporated into a polyvinyl chloride (PVC)-based membrane of a potentiometric electrode. In order to increase the lipophilicity and simultaneously the solubility of the ionophore in the PVC membrane [42,43], a decyl chain was incorporated into the macroring (see *rac*-**4** in Figure 1).

## 2. Experimental Section

### 2.1. Apparatus and Chemicals

Infrared spectra were recorded on a Bruker Alpha-T FT-IR spectrometer (Bruker Corporation, Billerica, MA, USA). ^1^H (500 MHz) and ^13^C (125 MHz) NMR spectra were obtained on a Bruker DRX-500 Avance spectrometer (Bruker Corporation, Billerica, MA, USA). Mass spectra were recorded on an Agilent-1200 Quadrupole LC/MS Instrument (Agilent, Santa Clara, CA, USA) using electrospray ionization. UV-Vis spectra were recorded on a UNICAM UV4-100 spectrophotometer controlled by VIZION 3.4 software (ATI UNICAM, Cambridge, UK). During spectrophotometric measurements, the aqueous solution of the metal salts (50 mM, 50 µL) was added to the ligand and dissolved in acetonitrile (0.1 mM, 2500 µL). All of the UV-Vis spectra were corrected by the blank sample prepared in the above way without the metal salts. Elemental analyses were performed in the Microanalytical Laboratory of the Department of Organic Chemistry, Institute for Chemistry, L. Eötvös University, Budapest, Hungary. Melting points were taken on a Boetius micro-melting point apparatus, and were corrected. Starting materials were purchased from Sigma-Aldrich Corporation (St. Louis, MO, USA, owned by Merck KGaA) and used without further purification, unless otherwise noted. Solvents were dried and purified according to well-established methods [44]. Silica Gel 60 F254 (Merck KGaA, Darmstadt, Germany) plates were used for thin-layer cromatography (TLC). Ratios of solvents for the eluents are given in volumes (mL/mL). Silica Gel 60 (70–230 mesh, Merck) was used for column chromatography. For potentiometric measurements, a Philips IS-561 (Glasblaserei Moller, Zurich, Switzerland) electrode body was used with an Ag/AgCl/3 M KCl // 1 M KCl double-junction reference electrode (Metrohm, Herisau, Switzerland) in a Radelkis OP-208/1 precision pH-meter (Radelkis Ltd., Budapest, Hungary) in all cases. 10^−3^ M lead(II) acetate was used as the inner filling solution during calibration, while in the selectivity measurements, strongly discriminated 10^−3^ M lithium(I) acetate was applied.

### 2.2. Synthesis of the Ionophore

**Scheme 1:**
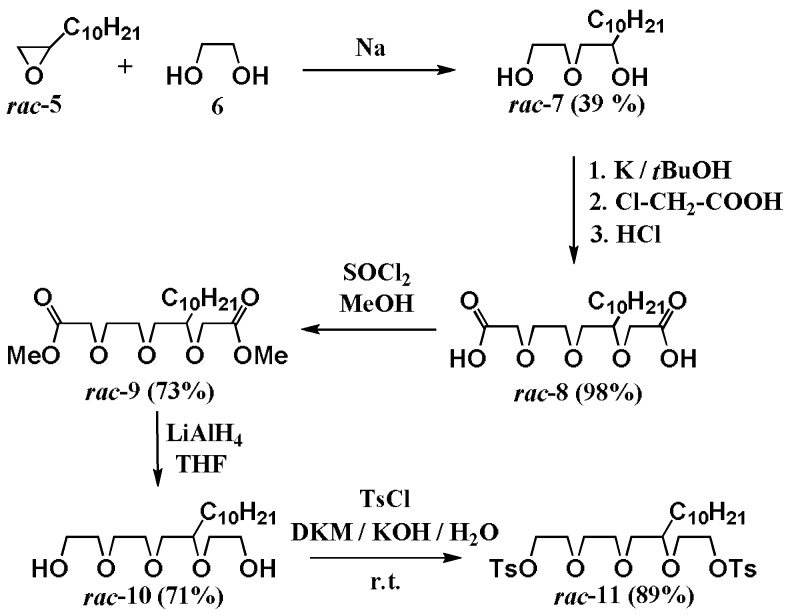
Synthesis of racemic, the lipophilic key intermediate *rac*-**11** of the new ionophore *rac*-**4**.

The synthesis of the lipophilic intermediate of the new racemic acridono-18-crown-6 ether that was used as a ligand in the ion-selective membrane was carried out as outlined in Scheme 1. The racemic intermediate *rac*-**10** was prepared according to the synthesis method of its pentaethylene glycol analogue [45]. The diol *rac*-**10** was reacted with 4-toluenesulfonyl chloride to form the ditosylate ester *rac*-**11**. The acridine-4,5-diol (**12**) was obtained by a reported procedure from 3-methoxy-benzoic acid [46].

**Scheme 2:**
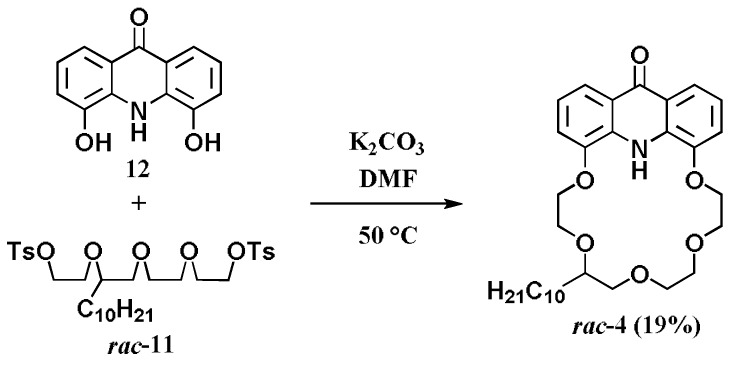
Macrocyclization of the new electroactive ligand *rac*-**4**.

The macrocyclization of acridine-4,5-diol (**12**) and the ditosylate *rac*-**11** in the presence of a weak base, potassium carbonate in *N*,*N*-dimethylformamide (DMF), resulted in the macrocycle *rac*-**4** (see in Scheme 2).

#### 2.2.1. 1-(2-Hydroxyethoxy)dodecan-2-ol [*rac*-**7**]

1,2-Epoxydecane (10.00 g, 54.30 mmol) was slowly added to a stirred solution of 0.50 g (22.22 mmol) sodium dissolved in 30.00 g (483.90 mmol) ethylene glycol at 150 °C under an Ar atmosphere. This mixture was stirred at 150 °C for two days, and the yellow reaction mixture turned brown. The mixture was cooled, and the excess ethylene glycol was distilled under reduced pressure. The residue was dissolved in 100 mL of dichloromethane and 50 mL of 6% aqueous sulfuric acid at 0 °C. The organic phase was separated and washed with dichloromethane (4 × 100 mL). The combined organic phase was shaken with 100 mL of sodium hydrogencarbonate and 100 mL of saturated brine, then dried over magnesium sulfate, filtered, and evaporated. The crude product was a viscous, light-brown oil, which was purified by column chromatography on silica gel using acetone:hexane (1:4) as an eluent to give *rac*-**7** (5.20, 39%) as a white solid. The physical properties of the purified product concurred with those in the literature [47].

M.p.: 48 °C; R_f_: 0.42 (SiO_2_ TLC, acetone:hexane 1:4); ^1^H-NMR (CDCl_3_): δ [ppm]: 0.87–0.91(t, *J* = 7 Hz, 3H); 1.27–1.31 (m, 15H); 1.41–1.48 (m, 3H); 3.34–3.36 (m, 1H); 3.38–3.55 (m, 1H); 3.59–3.78 (m, 2H); 3.80–3.83 (m, 3H); MS: Molecular weight calculated for C_14_H_30_O_3_: 246.22. Found *m*/*z*: 247.3 (M + H)^+^.

#### 2.2.2. 2-(2-{[2-(Carboxymethoxy)dodecyl]oxy}ethoxy)acetic Acid [*rac*-**8**]

Intermediate *rac*-**7** (4.50 g, 18.30 mmol) in 15 mL *t*-butyl alcohol was added to a stirred solution of 4.00 g (102.15 mmol) potassium in 120 mL of *t*-butyl alcohol at 80 °C under Ar. The mixture was refluxed for 1 h. Then, previously distilled chloroacetic acid (4.35 g, 45.75 mmol) in 15 mL of *t*-butyl alcohol was added dropwise over 30 min. The resulting mixture was stirred and refluxed for three days. The solvent was evaporated, and the residue was dissolved in 100 mL of water. The aqueous phase was washed with 200 mL of ethyl acetate. The pH was adjusted to 2, with concentrated HCl solution at 0 °C. The aqueous phase was shaken with ethyl acetate (3 × 200 mL). The combined organic phase was washed with 200 mL of saturated brine, and then dried over magnesium sulfate, filtered, and the solvent was evaporated under reduced pressure to give 6.50 g brown oil of the product *rac*-**8**. This compound was used without further purification to prepare *rac*-**9**.

#### 2.2.3. Methyl 2-(2-{[2-(2-methoxy-2-oxoethoxy)dodecyl]oxy}ethoxy)acetate [*rac*-**9**]

The dicarbonic acid *rac*-**8** (6.50 g) was dissolved in 80 mL of methanol. To the stirred solution, thionyl chloride (15.00 g, 125.50 mmol) was added dropwise over 2 h at 0 °C. The mixture was stirred at room temperature (r.t.) for 24 h; then, the methanol was removed under reduced pressure. The residue was dissolved in 100 mL of ethyl acetate, then washed with 50 mL of sodium hydrogencarbonate and 50 mL of saturated brine. The organic phase was dried over anhydrous magnesium sulfate. The solvent was evaporated, and the crude product was purified by column chromatography on silica gel adsorbent, using ethyl acetate:hexane (1:4) as eluent. The dimethyl ester *rac*-**9** (5.10 g, 73%) was a brown oil.

R_f_: 0.22 (SiO_2_ TLC, ethyl acetate:hexane 1:4); ^1^H-NMR (CDCl_3_): δ [ppm]: 0.89–0.92 (t, *J* = 7 Hz, 3H); 1.12–1.40 (m, 15H); 1.52–1.61 (m, 3H); 3.56–3.57 (m, 3H); 3.66–3.67 (m, 2H); 3.72–3.73 (m, 2H); 3.74–3.76 (s, 3H); 3.76–3.78 (s, 3H); 4.17–4.20 (m, 2H); 4.27–4.32 (m, 2H); MS: Molecular weight calculated for C_20_H_38_O_7_: 390.26. Found *m*/*z*: 391.3 (M + H)^+^.

#### 2.2.4. 2-(2-{[2-(2-Hydroxyethoxy)dodecyl]oxy}ethoxy)ethan-1-ol [*rac*-**10**]

Dimethyl ester *rac*-**9** (3.50 g, 8.96 mmol) was dissolved in 30 mL of THF. The solution was slowly added to a previously prepared, stirred mixture of 0.85 g (22.37 mmol) lithium aluminum hydride in 30 mL of THF at 0 °C under Ar. The resulting mixture was refluxed for three days. The mixture was cooled to 0 °C, and the excess reducing agent was hydrolyzed with a mixture of 1 mL of aqueous ammonium chloride (20 m/m%) and 2 mL of sodium hydroxide (20 m/m%). The resulting mixture was further stirred at r.t. for one day, then the precipitate was filtered and washed with diethyl ether (3 × 100 mL). The filtrate was separated. The aqueous layer was saturated with sodium chloride, and then extracted with diethyl ether (3 × 100 mL). The combined organic phase was washed with 100 mL of saturated brine, and then dried over magnesium sulfate, filtered, and the solvent was evaporated. The crude product was chromatographed on silica gel using ethyl acetate as an eluent. The diol *rac*-**10** (2.13 g, 71%) was a colorless oil.

R_f_: 0.31 (SiO_2_ TLC, ethyl acetate); ^1^H-NMR (CDCl_3_): δ [ppm]: 0.85–0.88 (t, *J* = 7 Hz, 3H); 1.12–1.30 (m, 18H); 3.31–3.39 (m, 3H); 3.50–3.55 (m, 4H); 3.66–3.70 (m, 3H); 3.87–4.05 (m, 4H); 4.09–4.22 (m, 1H); MS: Molecular weight calculated for C_18_H_38_O_5_: 334.27. Found *m*/*z*: 335.3 (M + H)^+^.

#### 2.2.5. 1-Methyl-4-[(2-{[1-(2-{2-[(4-methylbenzenesulfonyl)oxy]ethoxy}ethoxy)dodecan-2-yl]oxy}ethoxy)sulfonyl]benzene [*rac*-**11**]

To a stirred solution of *rac*-**10** diol (1.75 g, 5.24 mmol) in dichloromethane (15 mL) and potassium hydroxide solution (20 mL, 50 m/m%), 4-toluenesulfonyl chloride (3.00 g, 15.67 mmol) that had been dissolved in 15 mL of dichloromethane was added dropwise over 15 min at 0 °C. After addition, the mixture was stirred at r.t. for two days. The pH of the reaction mixture was adjusted to 7 with aqueous HCl solution (10 m/m%). The phases were separated, and the aqueous phase was extracted with dichloromethane (3 × 100 mL). The combined organic phase was dried over magnesium sulfate, filtered, and the solvent was removed under reduced pressure. The crude product was purified by column chromatography on silica gel using ethyl acetate:hexane (1:4) mixture as an eluent to get *rac*-**11** (2.98 mg, 89%) as a colorless, viscous oil.

R_f_: 0.75 (SiO_2_ TLC, ethyl acetate:hexane 1:1); ^1^H-NMR (CDCl_3_): δ [ppm]: 0.85–0.88 (t, *J* = 7 Hz, 3H); 1.24–1.35 (m, 18H); 2.45 (s, 6H); 3.35–3.41 (m, 3H); 3.49–3.54 (m, 4H); 3.64–3.68 (m, 3H); 3.77–4.10 (m, 1H); 4.11–4.14 (m, 4H); 7.25–7.33 (m, 4H); 7.77–7.79 (m, 4H); ^13^C-NMR (CDCl_3_): δ [ppm]: 14.29; 21.80; 21.80; 22.85; 25.56; 29.51; 29.74; 29.80; 29.88; 31.88; 32.08; 67.83; 68.87; 69.45; 69.88; 70.82; 70.90; 74.47; 79.78; 128.13; 129.95; 130.01; 133.18; 133.31; 133.31; 144.85; 144.99; IR (KBr) υ_max_: 2923, 2854, 1598, 1454, 1355, 1189, 1175, 1120, 1096, 1011, 917, 814, 772, 662, 553 cm^−1^; MS: Molecular weight calculated for C_32_H_50_O_9_S_2_: 642.29. Found *m*/*z*: 643.3 (M + H)^+^; Elemental analyses calculated for C_32_H_50_O_9_S_2_: C, 59.79; H, 7.84; S, 9.97. Found: C, 59.92; H, 7.81; S, 10.01.

#### 2.2.6. 10-Decyl-6,9,12,15,18-pentaoxa-25-azatetracyclo[21.3.1.0^5^,^26^.0^19^,^24^]heptacosa-1,3,5(26),19(24),20,22-hexaen-27-one [*rac*-**4**]

A mixture of acridine-4,5-diol (**7**) (0.30 g, 1.32 mmol), decyl-substituted tetraethylene glycol ditosylate *rac*-**6** (1.01 g, 1.58 mmol), finely powdered anhydrous potassium-carbonate (1.45 mg, 10.50 mmol), and dry DMF (50 mL) were stirred vigorously under Ar at r.t. for 10 min, then kept at 50 °C for a week. The solvent was removed under reduced pressure, and the residue was taken up in a mixture of ice water (75 mL) and ethyl acetate (150 mL). The phases were shaken well and separated. The aqueous phase was extracted with ethyl acetate (3 × 100 mL). The combined organic phase was dried over magnesium sulfate, filtered, and the solvent was evaporated under reduced pressure. The crude product was purified by column chromatography on silica gel using methanol:dichloromethane (1:50) mixture as an eluent to give *rac*-**4** (132 mg, 19%) as a white-yellow solid.

M.p.: >360 °C; R_f_: 0.75 (SiO_2_ TLC, methanol:dichloromethane 1:10); ^1^H-NMR (CDCl_3_): δ [ppm]: 0.79–0.82 (t, *J* = 7 Hz 3H); 1.17–1.47 (m, 18H); 3.43–3.62 (m, 2H); 3.65–3.95 (m, 9H); 4.17–4.34 (m, 4H); 7.10–7.20 (m, 2H); 7.40–7.90 (m, 2H); 8.20–8.30 (m, 2H); 9.85 (s, NH). ^13^C-NMR (CDCl_3_): δ [ppm]: 14.11; 22.69; 25.55; 25.71; 29.33; 29.58; 29.61; 29.71; 31.74; 31.92; 68.53; 69.27; 69.65; 70.44; 70.63; 71.41; 79.50; 79.81; 112.17; 112.53; 118.63; 118.69; 120.76; 120.81; 122.03; 122.03; 131.53; 131.58; 146.90; 147.09; 177.83; IR (KBr) υ_max_: 3421, 3070, 3031, 2924, 2851, 1626, 1609, 1533, 1489, 1447, 1360, 1268, 1223, 1120, 1081, 1071, 924, 821, 742, 692, 599 cm^−1^; MS: Molecular weight calculated for C_31_H_43_NO_6_: 525.31. Found *m*/*z*: 526.4 (M + H)^+^; Elemental analyses calculated for C_31_H_43_NO_6_: C, 70.83; H, 8.24; N, 2.66. Found: C, 70.93; H, 8.28; N, 2.69.

### 2.3. Preparation of Plasticized PVC Membranes

To incorporate the ligand *rac*-**4** into the potentiometric sensor, the following membrane composition was made: 1 mg of ionophore *rac*-**4**, 33 mg of PVC powder (Corvic S 704, ICI), 66 mg of 2-nitrophenyl-octyl ether (o-NPOE) polar plasticizer, or dioctyl sebacate (DOS) apolar plasticizer, and either one or two equivalents (regarding the ionophore) potassium tetrakis (4-chlorophenyl) borate lipophilic ionic additive were dissolved in 2 mL of THF. This solution was placed into a 20-mm diameter teflon ring. After evaporation of the solvent, a 7-mm diameter disk was cut out from the membrane and incorporated into the electrode body.

### 2.4. Potentiometric Measurement

All of the potentiometric measurements were carried out at room temperature. The electromotive force (EMF) of the cell was measured by varying the concentration of the stirred test solutions in the range 1.0 × 10^−7^ to 1.0 × 10^−1^ M by serial dilution. During calibration, the EMF values were recorded both with increasing and decreasing concentrations. Each point of the diagrams is from three independent measurements. The deviation was below ±0.5 mV in every case except for the measurements that were out of the working pH range, or over 20% organic content. Between two different sample solutions, the electrode pair was washed with distilled water, and then wiped off. All of the pH adjustments were made with concentrated HCl or NaOH. The response time of the membrane sensors—i.e., the time in which stable and constant potentials were recorded—was determined by measuring the potentials at different times. The potential values were recorded 10 s after immersing the electrode into the test solution.

Potentiometric selectivity coefficients were determined by the separate solution method based on the Nikolsky–Eisenman equation from the EMF data measured for metal (Li^+^, Na^+^, K^+^, Ag^+^, Mg^2+^, Cd^2+^, Zn^2+^, Co^2+^, Ca^2+^, Cu^2+^, Pb^2+^) acetate, Hg^2+^ chloride or perchlorate salts of protonated isopropylamine (iPrNH_3_^+^) and 1-(1-naphthyl)ethylamine (NEAH^+^).

Activity coefficients were calculated using the Debye–Hückel equation, EMF data were corrected by the diffusion potentials estimated with the Henderson equation.

## 3. Results and Discussion

### 3.1. Spectrophotometric Studies of Ionophore rac-***4***

**Figure 2:**
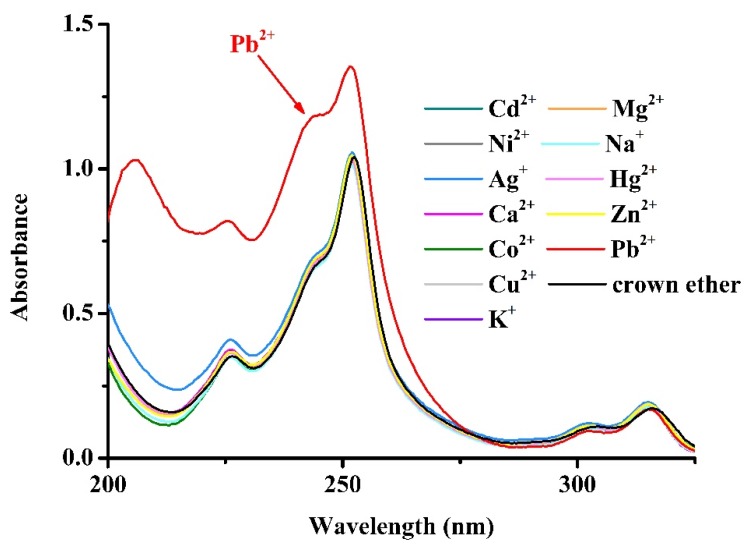
UV/Vis spectra of acridono-18-crown-6 ligand *rac*-**4** in the presence of 10 equivalents of metal salts.

Due to the presence of the chromophore acridone unit, the complexing ability of the ionophore *rac*-**4** could be studied using UV-Vis spectroscopy by adding different metal salts in a molar ratio of 10:1 to the solution of the ligand. As shown in Figure 2, the ionophore *rac*-**4** shows an increased absorbance only in the presence of lead(II) ions. The selectivity of macrocycle **3** (see Figure 1) toward various metal ions had been previously studied with the same method under the same conditions, and lead(II) ion selectivity was observed [41]. It can be concluded that the complex formation was not influenced by the introduction of the decyl group into the macroring of the parent crown ether **3** (Figure 2).

### 3.2. Potentiometric Characterization of the Electrode Membranes Containing Ionophore rac-***4***

Initially, the optimal composition of the membrane was determined following the literature. Focused on divalent ion selectivity, polar plasticizers are generally preferred [48]. The added lipophilic salt behaves as a cation-exchanger and provides permselectivity [49]. The optimal amounts of lipophilic additives depend on both the charge of the primary ion and the ionophore–ion complex stoichiometry [50,51,52]. First, the PVC based ion-selective membrane containing ionophore *rac*-**4** was plasticized with o-NPOE. Two equivalents (regarding the ionophore) of potassium tetrakis (4-chlorophenyl) borate were used.

**Figure 3:**
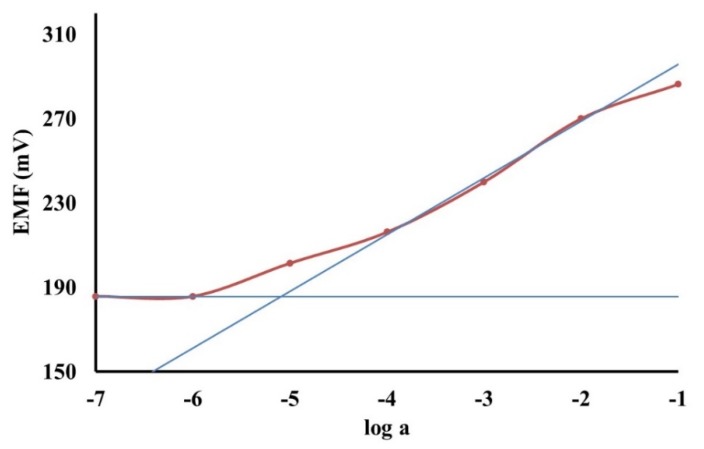
Calibration curve of the membrane electrode containing crown ether ionophore *rac*-**4** and 2-nitrophenyl-octyl ether (o-NPOE) as plasticizer with lead(II) acetate in aqueous solution.

The electrode was calibrated in different concentrations of lead(II) acetate solutions. The calibration curve is shown in Figure 3. The lower detection limit was determined according to the definition [48]. To the linear range of the calibration curve (marked with a red line in Figure 3), a linear regression line (marked with a blue line in Figure 3) was fitted. The coefficient of determination (*R*^2^) was over 0.99. The ordinate value of the intersection of the regression line and the constant potential (marked with a horizontal blue line in Figure 3) is the detection limit.

The electrode gives a near-Nernstian response to lead(II) ions with a slope of 26.9 mV/decade in the concentration range 10 × 10^−4^ to 10 × 10^−2^ M with a detection limit of about 7.9 × 10^−6^ M, and a low response time of 5 s. The deviation was below ±0.5 mV.

The selectivity of the electrode for various metal ions was assessed by recording the EMF response in the separate solutions of the measured and interfering ions. The difference in the EMF values was used to determine the slope of the calibration curve and calculate the potentiometric selectivity. The potentiometric selectivity coefficients reflect the ratio of the stability constants of the ionophore with the different ions. The potential response of the electrode was measured using 10^−3^ M solutions of metal ions and protonated primary amines. The potentiometric selectivity coefficients of the electrode for different interfering ions standardized on lead(II) ion are shown in Table 1.

We have found that the new ionophore *rac*-**4** showed good selectivity toward the lead(II) ion. The mercury(II) ion was the only one that interfered to a significant extent. The macrocycle *rac*-**4** showed significant preference to protonated primary amines, especially aralkylamines, over all the metal ions, including lead. These results are well correlated with former studies on crown ethers containing a heterocyclic unit, due to the strong л–л interactions between the aromatic moieties. Protonated aralkyl amines could be detected selectively in the presence of aliphatic analogues.

**Figure 4:**
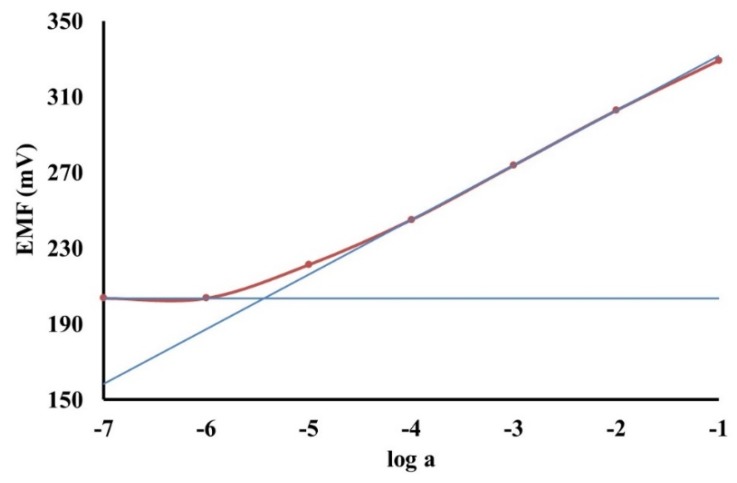
Calibration diagram of the PVC-based electrode membrane containing crown ether ionophore *rac*-**4** and dioctyl sebacate (DOS) plasticizer with lead(II) acetate.

The ionophore *rac*-**4** was also incorporated into a PVC membrane. In spite of the recommendation given in the literature [48,50,51,52], apolar plasticizer dioctyl sebacate and just one equivalent (regarding the ionophore) of lipophilic ionic additive were used. The calibration diagram can be seen in Figure 4. The calibration curve is indicated in red. The lower detection limit was determined according to the definition [48]. The *R*^2^ coefficient of the fitted regression line (marked with a blue line in Figure 4) was over 0.99.

The slope of the calibration curve was 28.9 mV/decade. The ideal operating range of concentration is 10 × 10^−4^ to 10 × 10^−2^ M, with a detection limit of about 4.0 × 10^−6^. The deviation was below ±0.5 mV.

The selectivity ratios toward monovalent and divalent ions measured using 10^−3^ M solutions of metal ions and protonated primary amines are presented in Table 2.

The results show that this ion-selective membrane composition was superior to the previous one with the o-NPOE plasticizer, as it showed a Nernstian response and the obtained selectivity values better; however, it has a slower response time of about 30 s. Modification of only one of the two investigated factors had no significant influence on the membrane characteristics.

### 3.3. Response and Lifetime

The PVC-based ion-selective electrode membrane containing *rac*-**4** crown ether as ionophore has similar operating characteristics as other lead(II) ion-selective liquid membrane electrodes reported in the literature [30,31,32,33,34,35,36,37,38,39,40], except that the membrane, containing o-NPOE plasticizer and two equivalents of lipophilic ion-exchanger, exhibits an outstandingly low response time of 5 s. Potentials remained constant for about 1 min, after which a slow drift was observed. The sensor molecule *rac*-**4** is chemically stable. During the application, the ionophore does not leach out from the membrane due to its extremely high lipophilicity. For different measurements (calibration, selectivity, determination, etc.), we did not use the same membrane to avoid disturbing effects. The same membrane was applied for up to 30 measurements. No change was observed in its characteristics during the investigations. The membrane could be stored over a period of three months without significant change in the calibration parameters.

### 3.4. Effect on pH and Adding Organic Solvents

**Figure 5:**
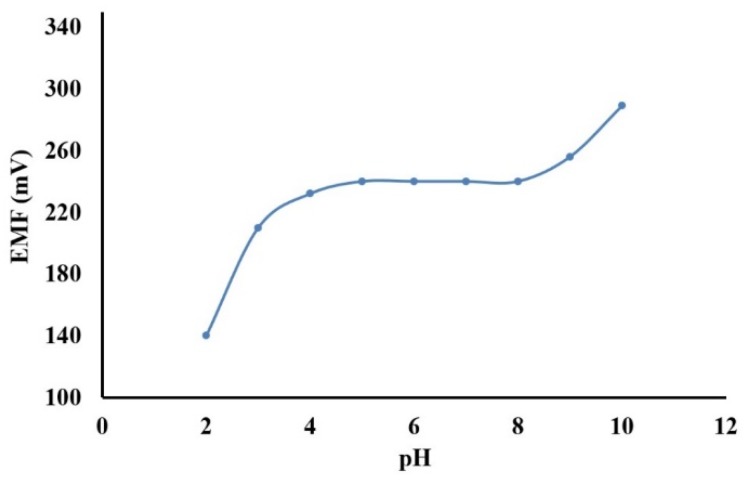
Effect of pH on cell potential using the electrode membrane containing o-NPOE plasticizer and two equivalents of lipophilic ion-exchanger, measured in aqueous lead(II) ion solution (10^−3^ M).

As the sensor is intended to be used in aqueous medium, further investigations were carried out to define the optimal pH range of the application. Therefore, the potential response of the electrode was measured in 10^−3^ M lead acetate solution while varying its pH. Figure 5 shows the effect of pH on the response.

Based on our results, the EMF is independent of the pH within the range of 4–8. We observed increasing potential values under basic conditions. In our opinion, this phenomenon can be explained by precipitation, which causes the lead(II) hydroxide adhesion to the membrane surface. Above neutral pH, the effects of precipitation have an influence on the potential response. Therefore, the electrode cannot supply reliable data regarding lead(II) ion concentration at pH > 7. It has been previously demonstrated [53] that a prototropic tautomerization equilibrium takes place during complex formation in the case of acridono-crown ethers. This equilibrium is probably not independent of the pH. Therefore, pH can also influence the electrode response by its effect on the sensor molecule. The upper limit of the working pH range is 7.

In more acidic medium, the electrode shows deviation in its behavior due to the protonation of the sensor molecule.

We have investigated the potential response of the electrode in aqueous/organic solvent mixtures as well at 10^−3^ M lead(II) acetate concentration. The application of the electrode in a non-aqueous medium is feasible below 20% organic content (investigated with acetone or methanol); above this limit, shifting of the potential values takes place.

### 3.5. Analytical Application of the Membrane Electrode under Competitive Conditions

In practical applications, it is hard to find monocomponent systems. Despite this, studies on applications under competitive conditions are rarely found in the literature. To demonstrate the utility of the sensor, further measurements were investigated by using the electrode under competitive conditions for a realistic multicomponent sample. Solutions contaminated by heavy metal ions usually contain various mineral salts, and the cations of these salts influence the results.

**Figure 6:**
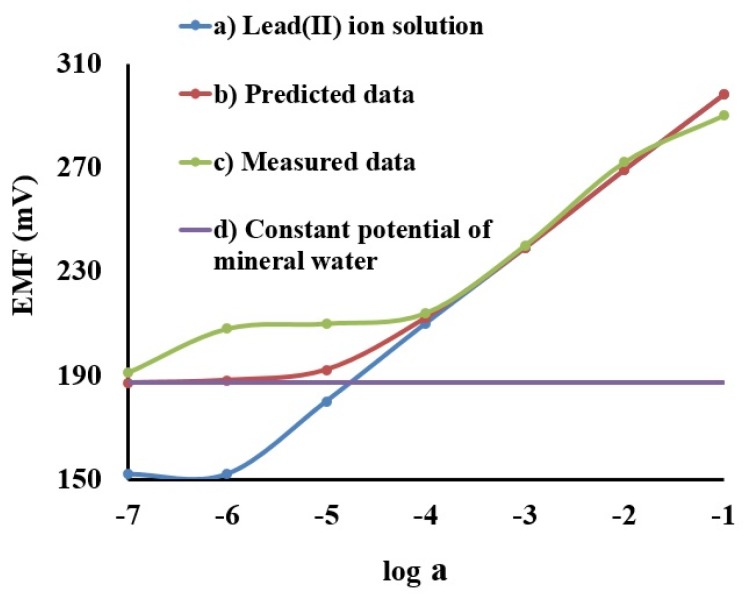
Application of the electrode membrane containing o-NPOE plasticizer and two equivalents of lipophilic ion-exchanger, under competitive conditions.

The chosen test sample was mineral water, which reportedly contained the following ions at these concentrations: Na^+^ (19 mg/L), Mg^2+^ (23 mg/L), and Ca^2+^ (59 mg/L). The mentioned concentrations are about 10^−3^ M, which concur with the functional concentration range of the electrode. During the experiment, lead(II) ion was added to the mineral water in different concentrations, and changes in the EMF were recorded. The results are shown in Figure 6, “Measured data”, curve (c). Curve (a) shows the calibration curve in the aqueous solution of lead, while line (d) indicates the potential response of the mineral water. The deviation was below ±0.5 mV in all of the cases.

It is possible to accurately predict the potential response of ion selective electrodes, even in mixtures of metal ions with different charges. The equation below describes the mixed ion response behavior of polymeric membrane-based ion-selective electrodes containing lipophilic ionophores and optimal amounts of other additives [54,55].

$$E_{m}=E_{I}^{0}+\frac{RT}{F}\ln\left[\frac{1}{2}\underset{i\left(1\right)}{\sum }K_{I,i\left(1\right)}^{pot^{\frac{1}{z_{1}}}}a_{i\left(1\right)}\left(aq\right)+\sqrt{{\left(\frac{1}{2}\underset{i\left(1\right)}{\sum }K_{I,i\left(1\right)}^{pot^{\frac{1}{z_{1}}}}a_{i\left(1\right)}\left(aq\right)\right)}^{2}+\underset{i\left(2\right)}{\sum }K_{I,i\left(2\right)}^{pot^{\frac{2}{z_{1}}}}a_{i\left(2\right)}\left(aq\right)}\right]$$

where the selectivity coefficient $K_{I,i}^{pot}$ is given by the following equation, where *I* represents the primary ion, and *i* represents the monovalent and divalent interfering ions.

KI,ipot=KIKizIzj

We have used this equation to predict the response of our electrode in the mineral water at different added concentration levels of lead(II) ion. The obtained values are plotted in Figure 6 curve (b). The predicted response is in a good agreement with the measured values in the concentration range between 10^−4^ and 10^−2^ M. The response of the electrode containing the optimized membrane is predictable within a 5% error limit with the equation above in the working concentration range.

In the concentration range of 10^−4^ to 10^−2^ M, the sensor is suitable for the selective and quantitative analysis of lead(II) ions, despite the presence of interfering ions.

## 4. Conclusions

We have synthesized and characterized a new crown ether *rac*-**4** with the aim of using it as an electroactive ionophore in potentiometric sensing. We have demonstrated by UV-Vis spectroscopy that the presence of the decyl chain attached to the macroring had no influence on complexation of the macrocycle.

The new sensor molecule was incorporated into a plasticized PVC membrane using different membrane compositions. The linearity and the selectivity of the ion-selective electrode toward different metal and protonated primary amines were investigated. The values of the selectivity coefficient show that the electrode is highly selective to lead(II) ion, even in the presence of potentially interfering metal ions, but altogether has a significant preference for protonated primary amines. The linear concentration range is from 10^−4^ to 10^−2^ M, and the working pH range is from 4 to 7. The allowed upper limit of organic solvents in the solutions of samples is 20%. Under these conditions, the sensor is able to perform selective and quantitative analysis of lead(II) ions with a low response time of 5 s. The applicability of the sensor for the quantitative determination of lead has been demonstrated in mineral water, which is a multicomponent sample containing relatively high levels of possible interfering ions.

## Figures and Tables

**Table 1:** Potentiometric selectivity of the polyvinyl chloride (PVC)-based electrode membrane containing ligand *rac*-**4** and o-NPOE as the plasticizer for different metal ions and protonated primary amines.

Ions	logK_Pb,j_
Li^+^	−4.60
Na^+^	−4.53
K^+^	−3.86
Ag^+^	−3.45
Mg^2+^	−2.45
Cd^2+^	−2.34
Zn^2+^	−2.30
Co^2+^	−2.27
Ca^2+^	−2.04
Cu^2+^	−2.04
Hg^2+^	−0.56
Pb^2+^	0.00
iPrNH_3_^+^	2.31
NEAH^+^	8.84

**Table 2:** Potentiometric selectivity of the PVC-based electrode membrane containing ligand *rac*-**4** and DOS as a plasticizer for different metal ions and protonated primary amines.

Ions	logK_Pb, j_
Li^+^	−5.11
Na^+^	−4.98
K^+^	−4.39
Ag^+^	−4.08
Mg^2+^	−2.91
Zn^2+^	−2.91
Co^2+^	−2.87
Cd^2+^	−2.80
Ca^2+^	−2.56
Cu^2+^	−2.46
Hg^2+^	−0.83
Pb^2+^	0.00
iPrNH_3_^+^	3.49
NEAH^+^	10.51
